# Corrigendum to: microRNAs as novel antidepressant targets: converging effects of ketamine and electroconvulsive shock therapy in the rat hippocampus

**DOI:** 10.1093/ijnp/pyad002

**Published:** 2023-02-03

This is a corrigendum to: Richard M. O’Connor, Susan Grenham, Timothy G. Dinan, John F. Cryan, microRNAs as novel antidepressant targets: converging effects of ketamine and electroconvulsive shock therapy in the rat hippocampus, *International Journal of Neuropsychopharmacology*, Volume 16, Issue 8, September 2013, Pages 1885–1892, https://doi.org/10.1017/S1461145713000448

In November 2022, the corresponding author reported to the journal that some of the fold changes reported in Figure 1D did not match the raw data and, after review, found that the columns had been mislabeled due to a sorting error. When comparing the published and corrected figures (below), the labels have changed but the magnitude of the columns remain the same. Additionally, the visual representation of the fold change of 4 miRNAs was reported as being opposite to the actual direction induced by antidepressant treatment, namely miR-487b, mir-222, mir-327 and mir-129.

Published Figure 1D


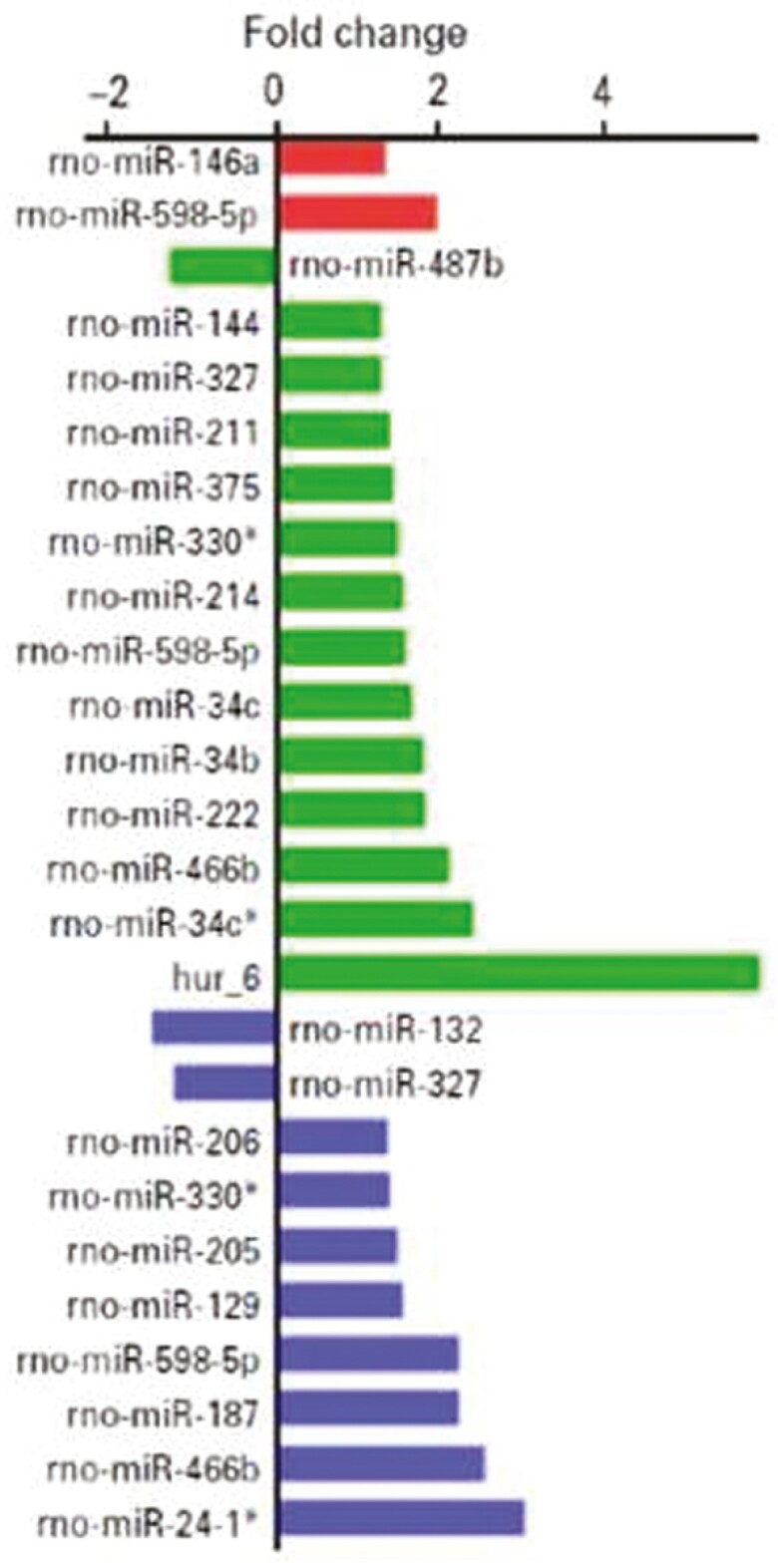


Revised Figure 1D


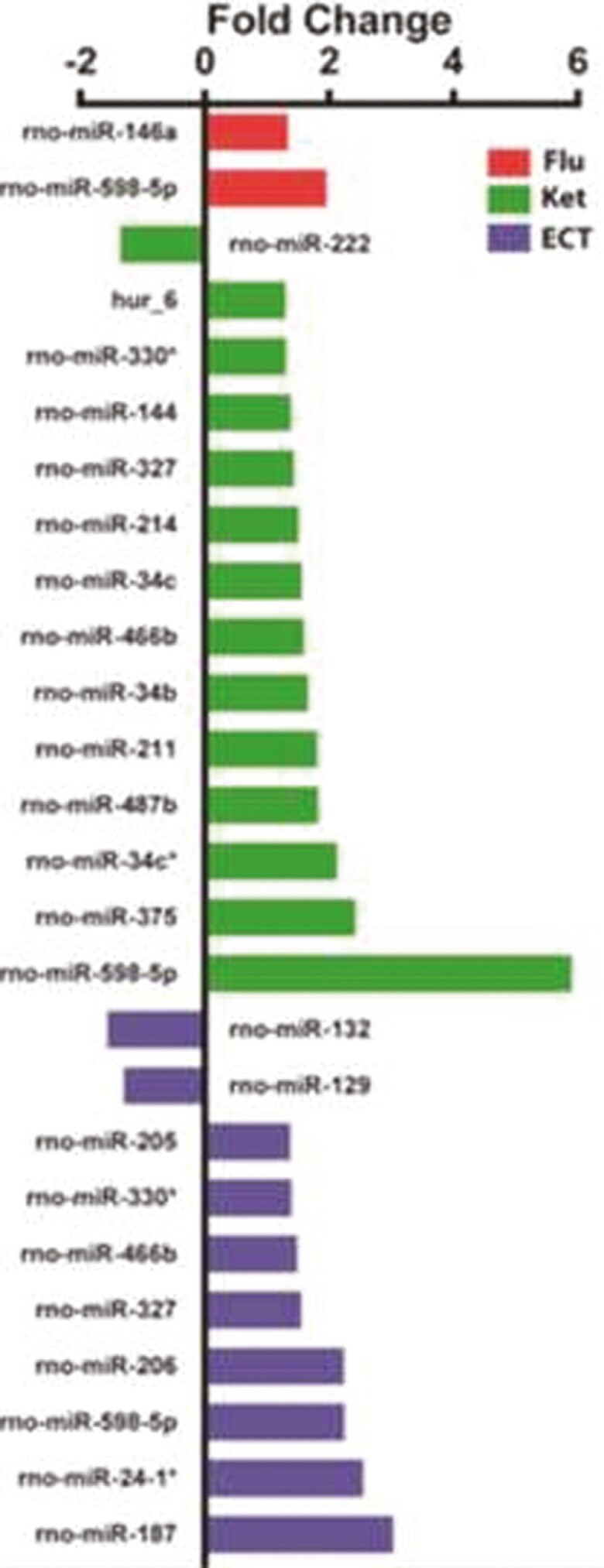


Both the authors and editors confirm these errors do not change the overall conclusions and the data presented elsewhere in Figure 1 and in Figure 2 remain unaltered. The authors apologize for these unintentional errors.

These details have been corrected only in this correction notice to preserve the published version of record.

